# An Historic Annual Meeting

**Published:** 1919-05-10

**Authors:** 


### KING EDWARD'S HOSPITAL FUND FOR LONDON.

### An Historic Annual Meeting.

THE annual meeting of the President and General Council of King Edward's Hospital Fund for London, to receive the accounts and the report for the year 1918, was held at St. James's Palace on Tuesday morning, May 6, H.R.H. the Prince of Wales, President of the Fund, being in the chair.

Those present were :—The Rev. W. L. Watkinson, D.D., the Marquess of Cambridge, the Viscount Knutsford, the Viscount Mersey, the Viscount Sandhurst, the Viscount Farquhar, the Viscount Burnham, Lord Revelstoke, Lord Stamfordham, Lord Somerleyton (Hon. Secretary), Lord Stuart of Wortley, the Right Hon. James W. Lowther, M.P. (Speaker of the House of Commons), the President of the Hospital Sunday and Hospital Saturday Funds (the Right Hon. the Lord Mayor), the Chairman of the County Council (the Lord Downham), the President of the Royal College of Physicians (Sir Norman Moore, Bart.), the President of the Royal College of Surgeons (Major-General Sir George Makins), the Right Hon. Sir T. Vezey Strong, Sir William S. Church, Bart., Sir Francis H. Champneys, Bart., Lieut.-General Sir Alfred Keogh, Sir Owen Philipps, M.P., Sir Henry Burdett, Sir William J. Collins, Sir Cooper Perry, Sir John Craggs, Sir John Tweedy, Mr. Frederick M. Fry (Hon. Secretary), Mr. John G. Griffiths (Hon. Secretary), Mr. Yvon R. Eccles, Mr. Geo. Lawson Johnston.

### THE YEAR'S SURPLUS INCOME.

**Lord Revelstoke** (the Honorary Treasurer), in presenting the account of receipts and expenditure and the balance sheet for the year ending December 31, 1918, said that the Council might remember that when it was decided last autumn to increase the distribution from £190,000 to £200,000, the estimated income for the year, after allowing for expenses, fell short of that sum by about £6,000. During the last fortnight of December, however, they received an unexpected legacy, with the result that instead of a deficit there was a surplus of nearly £3,000.

With regard to investments of the Corporation, the position might be regarded as satisfactory. The market value of the investments of the Fund at the end of 1918, as compared with the valuation made in 1908 upon the passing of the Act of Incorporation, showed a net depreciation of 10.72 per cent. after allowing for appreciation and depreciation realised since the year 1908. He might add that more than 63 per cent. of the invested funds were in the form of British Government Securities.

**The Prince of Wales** formally moved the adoption of the accounts.

The resolution, having been seconded by the **Lord Mayor,** was then put and carried unanimously.

### The Council's Annual Report.

**Mr. Frederick M. Fry** (Hon. Secretary) presented the draft report of the Council for the year 1918, as follows :—

### THREE ROYAL PRESIDENTS.

1. The General Council desire to record the gratification with which they have learned that His Majesty the King, Patron of the Fund, has, pursuant to King Edward's Hospital Fund for London Act, 1907, appointed His Royal Highness the Prince of Wales President of the Fund. This office was held by his grandfather King Edward VII. from 1897 to 1901 and by His present Majesty from 1901 to 1910, and the Fund has throughout its career largely benefited by the active assistance of its Royal Presidents. The General Council desire to express their warm appreciation of the services of the Marquis of Cambridge, Viscount Iveagh, and the Speaker of the House of Commons, who during the past nine years have, as Governors of the Fund, exercised so efficiently the powers of President.

### TOTAL RECEIPTS FOR THE YEAR.

2. The total receipts for the year 1918 were £218,679 15s. 4d., of which £12,160 2s. 7d. were contributions to Capital and £206,519 12s. 9d. receipts on General Account.

3. General Fund receipts were made up as follows :

| | £ | s. | d. |
|---|---|---|---|
| Annual Subscriptions ... ... | 25,254 | 2 | 9 |
| Donations ... ... ... ... | 6,902 | 16 | 3 |
| League of Mercy ... ... ... | 16,000 | 0 | 0 |
| Legacies ... ... ... ... | 44,460 | 3 | 8 |
| Dividends and Interest from Investments ... ... ... | 113,702 | 10 | 1 |
| Trustees of the Bawden Fund ... | 200 | 0 | 0 |
| Making a total of | £206,519 | 12 | 9 |

### A Continuous Growth in Distribution.

4. The Fund, founded by His late Majesty King Edward VII., then Prince of Wales, in 1897, celebrated its coming of age in 1918 by distributing for the first time a sum of £200,000. A total of £100,000 was first reached in 1902, and £150,000 in 1909. The amount distributed in 1917 was £190,000 and in the year before the war £157,500.

5. Of the amount distributed £190,000 was given to London hospitals and £10,000 to consumption sanatoria and convalescent homes taking London patients, as against £181,000 and £9,000 respectively in 1917.

### HOW THE GRANTS WERE ALLOCATED.

6. The £190,000 granted by the Fund to London hospitals was applied as to £144,450 in aid of general maintenance, £15,800 to the reduction of debts on maintenance account, £10,100 towards improvement schemes undertaken before the war or in reduction of liabilities on such schemes, and £19,650 in aid of new schemes. Grants for maintenance amounted to £16,825 more than in 1916, or £8,075 more than in 1917.

7. Of the £10,000 distributed by the Convalescent Homes Committee £7,825 was allocated to consumption sanatoria and £2,175 to convalescent homes. The grants to sanatoria enabled 64 beds to be reserved for the use of patients in London hospitals as compared with 62 last year.

### AVERAGE ANNUAL DISTRIBUTION £120,837.

8. The total sum distributed amongst hospitals, convalescent homes and consumption sanatoria during the last ten years (after deducting conditional grants to the extent of £2,500 that have allowed to lapse) is £1,674,500. Since the foundation of the Fund a total amount of £2,658,416 8s. 5d. has been distributed. The average of the 22 distributions during the whole period has been £120,837 2s. 6d.

### ADMINISTRATION LESS THAN 3D. IN EVERY £1.

9. During the year the amount spent on the administration of the Fund was £3,672 19s. 2d., or £1 13s. 7d. per

**King Edward's Hospital Fund—(*continued*).**

£100 of the total amount received, as compared with £3,460 10s., or £1 9s. 0½d. per £100 in the previous years. The corresponding percentage for the whole period since the inauguration of the Fund has been £1 4s. 11d., or less than 3d. in every £1 received, the average yearly outlay in this respect being £2,902 10s. 11d. The increased expenditure in 1918 has been occasioned partly by additions to salaries of the staff, and also by the greater cost of stationery, printing, etc., caused by the war, and by the cost of the Pensions Report.

**Royal and Other Subscriptions.**

10. His Majesty the King, Patron of the Fund, has been graciously pleased to continue his annual subscription of £1,000. Her Majesty the Queen, Her Majesty Queen Alexandra, and His Royal Highness the Prince of Wales have also been pleased to subscribe to the Fund, as have also Their Royal Highnesses Prince Albert, Princess Mary, and many other Members of the Royal Family

11. The list of contributions again includes annual subscriptions of £5,000 from Mr. Walter Morrison, £4,000 from the Drapers' Company, £1,000 from Viscount Astor, and £1,000 from the Merchant Taylors' Company. The Fund has also received annual subscriptions of £525 from Messrs. Lloyds Bank, Limited (in addition to a special donation of £525), and of £500 from Viscount Iveagh, from Sir John R. Ellerman, Bt. (in addition to a special donation of £500), from Mr. Robert Fleming, from Messrs. Morgan, Grenfell & Co., from Messrs. N. M. Rothschild & Sons, and from the family of the late Sir S. Neumann, Bt.; donations of the same amount from the Clothworkers' Company, from "S.D.R.S.D.," and from Messrs. John Swire & Sons, Limited; besides an anonymous gift of £3,000 India 3½ per cent. stock.

League of Mercy's Twenty Years' Work.

12. The League of Mercy this year has contributed £16,000, a sum greater by £1,000 than was received from the League last year. The total amount contributed to the Fund by the League of Mercy since its foundation in 1899 now amounts to £276,000, apart from the sums awarded by the League to institutions outside the area of the Fund's distribution. The greatest credit is due to the large number of voluntary workers who have maintained the contributions of the League of Mercy at so high a level during a period of exceptional difficulty.

13. The increased distribution was rendered possible mainly by an increase in the amount received from legacies. To maintain an annual distribution of £200,000 about £90,000 has to be raised from this source and from annual subscriptions and donations.

The Year's Legacies.

14. Legacies received during the year included £20,000 from the estate of the late Brigade-Surgeon John Law, on account of residue; £6,802 8s. 10d. from the estate of the late Sir Julius Charles Wernher, Bt., further on account of residue left to capital; £5,254 6s. 3d. from the estate of the late Mr. N. J. Wilson Blackett, on account of residue; £3,173 11s. 1d. from the estate of the late Mr. Alfred John Heath, being a final payment in respect of residue left to capital; £5,000 from the estate of the late Madame L. G. Oumiroff; and £5,000 given to the fund by the executors of the late Mr. Thomas Stephen Whitaker, in the exercise of discretionary powers. The fund also received £5,069 16s. 10d. under the will of the late Mr. George Herring, the proceeds of the winding-up of the Salvation Army Small Holdings Scheme, which was established by him with reversion to the fund.

Large Additions to Hospitals' Maintenance Cost.

15. The Statistical Report published by the Fund on the expenditure of London hospitals showed that the average number of beds in daily occupation by naval and military patients increased from 2,855 in 1916 to 3,656 in 1917. The total ordinary expenditure of the hospitals had increased to £1,719,030, or £580,500 more than in 1913. This increase was partially covered by contributions from the authorities towards the treatment of naval and military patients, amounting to £268,960. The balance, amounting to £311,540, represents the additional ordinary expenditure which fell on the funds of the voluntary hospitals of London during 1917, an increase of 27.36 per cent. on the expenditure in 1913. These large additions to the cost of maintaining the London hospitals are a sufficient proof of the increased claim of hospitals on the Fund and on the public.

**Pensions for Hospitals' Officers—Serious Defects.**

16. The inquiry into the question of pensions for hospital officers, begun by a sub-committee of the Executive Committee in 1914, but suspended during the early part of the war, was completed in 1918. The inquiry was held at the request of the Hospital Officers' Association and with the consent of the hospitals. The sub-committee presented a report signed by Mr. W. J. H. Whittall, Chairman, and Mr. H. L. Hopkinson, with a dissentient memorandum signed by Sir William Collins. The sub-committee are agreed that, except at a few larger hospitals which have schemes, there is great uncertainty and lack of uniformity in dealing with pensions to retiring officers, that these and other serious defects are detrimental both to the officers and to the hospitals, and that they can only be effectually remedied by a general scheme covering the whole of the hospital service.

After a survey of existing methods of providing pensions in various other services, the report lays down the general principles which should be observed in any scheme for hospital officers. These include financial provision in advance by contributions from the hospitals and the officers, and the full maintenance of pension rights in the event of migration from one hospital to another within the scheme.

No Guarantee in Aid.

On the question of the best method of obtaining the desired conditions the Sub-Committee are not unanimous, and the Executive Committee, after considering the reports, passed the following resolutions:

(1) That this report, with the Dissentient Memorandum and appendices, be published for the consideration of the parties concerned.*

(2) That the Committee cannot recommend to the Governors and General Council that King Edward's Hospital Fund should, in respect of its general funds, give any guarantee in aid of any pension scheme.

The Council are greatly indebted to the three members of the Executive Committee who carried out this inquiry, and commend the report to the consideration of the hospitals.

17. Every eligible hospital which applied for a grant was inspected and reported upon, as hitherto, by two of the Fund's visitors, one medical and the other lay. Several lady visitors were for the first time appointed. The inspection of consumption sanatoria was again prevented by the restrictions on travelling. All the sanatoria receiving grants had, however, already been visited for several successive years.

* The report was published in March 1919.

**King Edward's Hospital Fund**—(*continued*).

THE MINISTRY OF FOOD'S REQUEST.

18. At the beginning of 1918 the Ministry of Food were desirous of obtaining the services of Mr. H. R. Maynard, and the Fund readily agreed, with his concurrence, to an arrangement by which his time was divided between the Ministry and the Fund. This arrangement is still in force, and has thrown a heavy burden on him and on the other members of the staff. The Council desire to acknowledge the conscientious manner in which the work of the Fund has been carried on by all concerned.

DEATH AND RETIREMENT.

19. The Council regret to record the death of Dr. Freshfield, Chairman of the Convalescent Homes Committee since 1915. Dr. Freshfield had been a member cf the Committee since 1898, and of the Council since 1905, and had also rendered great services as head of the firm cf Messrs. Freshfields, honorary solicitors to the Fund since its foundation.

The Council have also to regret the retirement of Sir William Church from the Distribution Committee, of which he had been Chairman for sixteen years. The Council are deeply grateful for his great services during this period, and trust that he may long be able to continue his connection with the Fund as a member of the Council.

Sir John Tweedy has been appointed Chairman of the Distribution Committee, and Sir William Bennett of the Convalescent Homes Committee.

The Council regret that Mr. Walter Morrison has been unable to continue his membership of the Council.

20. The President of the Local Government Board, in pursuance of his powers under the Act, has again appointed Messrs. Deloitte, Plender, Griffiths & Co., chartered accountants, to audit the accounts of the Fund for the year. They have been the honorary auditors since January 1898, during which period they have rendered the Fund very valuable service.

21. The Council are under great obligations to the Press for the assistance they have rendered in the past, and continue to rely upon their kindly co-operation in obtaining for the Fund wider publicity and support.

*St. James's Palace* : May 6, 1919.

### HIS ROYAL HIGHNESS'S SPEECH.

**The Prince of Wales,** who on rising to move the adoption of the report was warmly received, said :—

My Lords and Gentlemen,—It is with a deep sense of responsibility that I have accepted the duties, laid upon me by command of His Majesty the King, of President of this great Fund. I would remind you that it was founded twenty-two years ago by my grandfather, King Edward VII., who was its President for four years; my father was President for nine years, and on his accession became its Patron. I count it a great honour to be called upon to carry on their work, a work dear to them both and which has proved so beneficial to the hospitals of London. (Hear, hear.)

Under their continuous personal supervision the Fund secured that position in the confidence of the public which it still holds. By the time that King Edward ceased to be President and became Patron the annual distribution had risen to £42,000, and when my father ceased to be President it had reached £150,000 The constitution, the methods and the traditions which they established have stood the test of time, and it will be my earnest endeavour, as your President, to adopt and to develop the principles which they have so well laid down. (Hear, hear.)

His Majesty desires me to express his thanks, to which I wish to add my own, to Lord Cambridge, Lord Iveagh, and the Speaker of the House of Commons, who, as Governors of the Fund, have so ably carried out the duties of President since 1910, during my minority and also during my absence on active service overseas. My first act was to appoint them members of the General Council, and I hope the Fund may long continue to have the benefit of their experience. (Hear, hear.)

It is gratifying to know that the finances of the Fund are in such a satisfactory condition in spite of the exceptional difficulties of the present time. The distribution last year reached for the first time the sum of £200,000, and the whole of this was provided out of the receipts for the year. We are greatly indebted to Lord Revelstoke and the Finance Committee for the care and success with which they have managed the investments. (Hear, hear.) The income from these investments is the solid foundation for what I hope may prove an ever increasing distribution. But in order merely to maintain the latter at its present level we must rely on the public for about £90,000 from annual subscriptions, donations and legacies, and any increase in the distribution must therefore depend on the increased receipts from these sources.

That the money is well spent is shown by the figures quoted in the annual report. In addition to their work for civilian patients, the hospitals of London had, on the average, 3,656 beds occupied by sailors and soldiers last year. Their expenditure in 1918, after allowing for the payments from the Admiralty and the War Office, was £300,000 more than in 1913, and over and above this they will now have to face the cost of repairs, of renewals and of improvements that have been necessarily postponed during the war. They will therefore need all the help that the King Edward's Hospital Fund can give.

The report on Pensions for Hospital Officers, prepared by a Sub-Committee of the Executive Committee, is an important addition to the Fund's list of publications on hospital matters. It is full of information about hospital pensions, as they are now and as the Sub-Committee considers they should be, and about pension schemes generally, much of which is likely to be of interest to all who are concerned with pension problems. The Sub-Committee were convinced that at the great majority of hospitals the existing provision for pensions was seriously defective, and that a satisfactory general scheme would not only prevent hardship to individuals, but would raise the status and increase the efficiency of the whole service, and, by so doing, would benefit the hospitals themselves in their work for the sick poor. An adequate pensions scheme is thus recognised as a legitimate and even a profitable branch of hospital expenditure, and from this point of view the conclusions of the report may appeal to donors who are specially interested in the efficient organisation of the hospital service of London.

Our best thanks are due to the members of the Sub-Committee, and particularly to the Chairman, Mr. Whittall, who has devoted a very great amount of expert labour to the inquiry and the preparation of the report. (Hear, hear.)

To all who have so generously given their services or other support to the King Edward's Hospital Fund I tender my hearty acknowledgments and grateful thanks.

I now move the adoption of the annual report of the Council for the year 1918. (Hear, hear.)

King Edward's Hospital Fund—(*continued*).

**The Marquis of Cambridge** having seconded the resolution, the adoption of the report was then put and carried unanimously.

Congratulations to the Prince.

**The Speaker of the House of Commons,** in moving a vote of thanks to H.R.H. the Prince of Wales for presiding, said:

My Lords and Gentlemen,

I am sure that I shall be voicing the views of everybody here present to-day if I ask you to pass a hearty vote of thanks to His Royal Highness the Prince of Wales for having taken the chair. I am sure also that you will desire to congratulate him upon his return from the Front safe and sound after all the experiences which he has gone through, and upon the promptitude and readiness with which he has at once taken up the onerous duties awaiting him here at home. Amongst those duties, though not amongst the most onerous, is that of being President of King Edward's Hospital Fund for London. He was kind enough to refer to the services which have been rendered by Lord Cambridge, Lord Iveagh, and myself during the time of his minority and during his absence abroad. Speaking for myself, and I am sure on behalf of my two colleagues, I can only say that it has been a labour of love, and that we have been very pleased to render all the services that we could during His Royal Highness's absence. I will not say anything further on this occasion, but, on behalf of the Council, express our very great pleasure at seeing His Royal Highness amongst us, and the hope that during the years that he presides over us the advance in the utility of the Fund may be as rapid and as great as that which took place during the time that his father, His Most Gracious Majesty, occupied the chair which he now occupies. I have much pleasure in proposing a hearty vote of thanks to His Royal Highness for presiding to-day.

**Sir Alfred Keogh,** in seconding the resolution, said he was sure they would all desire to associate themselves with the remarks of the Speaker. It was unfortunately true that the cost of the maintenance of sailors and soldiers in the London hospitals during the war had thrown a great burden on the Fund, but he had no doubt that, in the work of the coming years the active association with the Fund of His Royal Highness the Prince of Wales would be of inestimable value.

The resolution was then put and carried by acclamation.

The President's Thanks.

**His Royal Highness** said: My Lords and Gentlemen, I thank you very sincerely for the kind way in which you have passed this vote of thanks. It is a very great pleasure to me now to take over my duties as President of this Fund, and to have the advantage of the help of so many distinguished people who have been working at it so hard and for such a long time.

The proceedings then terminated.

